# To Report or Not to Report? A Qualitative Analysis of Journalists’ Perspectives on Harm to Public Opinion

**DOI:** 10.1093/poq/nfaf028

**Published:** 2025-08-07

**Authors:** Ricardo R Ferreira, Jean-François Daoust

**Affiliations:** Guest Lecturer, School of Social and Political Science, University of Edinburgh, Edinburgh, Scotland, UK; Associate Professor, School of Applied Politics, Université de Sherbrooke, Sherbrooke, QC, Canada

## Abstract

Journalists face intricate decisions regarding what to publish, especially when problematic content may impact public opinion in a way that could fuel hate and/or undermine democratic attitudes. While scholarship has recognized the importance of this issue, most studies focus on published content, how citizens engage with it, and the implications of published news. In this article, we provide a fresh perspective on the crucial dilemma faced by journalists concerning their perceived impact on public opinion, by leveraging data based on 36 semistructured in-depth interviews with journalists covering Brazil’s political landscape. The interviews were conducted between December 7, 2021, and July 20, 2022. Our main findings are threefold. First, we find a consensus among journalists regarding what is seen as problematic content, which is centered around threats to democratic attitudes and misinformation on critical issues. Second, we examine the rationales underpinning journalists’ choices to publish problematic content, which include the concept of “competing voices,” the legitimacy conferred to elected representatives (e.g., the head of a government), and journalists’ fear of being viewed as left leaning and losing their audience. Third, we find that journalists who do not publish problematic content do so because they expect to negatively impact public opinion, in particular democratic attitudes, and that their reporting of hate speech may not meet ethical standards. We conclude by highlighting the complex interplay of journalistic norms and expectations regarding their impact on public opinion and the news production process.

News media are crucial for the functioning of democracy. Among other things, reporters provide political information that will inform public opinion which, in turn, will inform citizens’ evaluation of their government (Mughan and Gunther 2012; Voltmer and Rawnsley 2019). Such assessments are critical for holding elected representatives accountable.

But what kind of information should the news media report on? News organizations regularly face a dilemma between covering stories that might be useful for citizens and the potential risks of fueling hate or exacerbating social tensions and polarization through this coverage. On the one hand, journalists are expected to hold public officials accountable for their actions and statements, which implies reporting on their socially undesirable actions or statements, including hate speech. On the other hand, such information may cause more harm than good for society, since it may negatively impact public opinion, especially concerning democratic attitudes in contexts of democratic instability or backsliding.^1^ Journalists must therefore weigh the value of informing the public against the potential harms caused by problematic events or statements that would be covered.

Given the importance of this dilemma for the democratic role of news organizations, scholars have analyzed their news-sharing behavior within this context. This scholarship mostly engages in quantitative analyses that use, among other things, content and text analyses (for recent examples, see Aruguete, Calvo, and Ventura 2021; Brown and Mondon 2021; Sbaraini Fontes and Marques 2023), which provide a thorough description of what gets published and how readerships interact with different types of news in various contexts. However, this strand of research investigates neither *journalists’* perceptions of what is potentially problematic content, nor their rationale behind whether to publish something or not. This gap is important and must be addressed for several reasons.

(1) Journalists’ norms and practices are important because they shape news outputs, which can influence public opinion. Journalists select, assign value to, and hierarchize information, including frames and sources (O’Neill and Harcup 2009; Shoemaker, Vos, and Reese 2009). (2) While recent research showed that citizens turn to legacy news media in a context of uncertainty or conflict (Langer and Gruber 2021; Newman et al. 2021), decision-making on what and how to publish is even more relevant vis-à-vis an increasing volume of information available across multiple platforms. (3) It is likely that, in contexts of democratic backsliding and where populist leaders have gained popularity, the democratic role of news organizations will become even more important. As such, the manner in which journalists deal with their audience and their potential effect on public opinion becomes even more significant in such contexts.

News outputs are powerfully shaped by institutional structures, such as regulatory framework, market characteristics, professional and organizational guidelines, the availability of resources, and journalists’ agency and professional judgement (Cook 1998; Gitlin 2003; Deuze 2008). Journalists’ decisions are also influenced by their personal positioning, including their sociodemographic background, political views, and life conditions (Deuze 2002, pp. 41–43). Moreover, these actions can be further influenced by their perceptions of how readers and viewers might react to the content and, in some cases, the reactions of the audiences that reach them.

In this research, we provide a fresh approach to studying how journalists’ views of public opinion shape their norms and practices, and, ultimately, their democratic functions. To do so, we leverage extant qualitative data from 36 semistructured in-depth interviews with journalists reporting on Brazilian politics for the country’s top three news organizations, focusing on the 2018 presidential election campaign (January to October 2018) and the first three years of Jair Bolsonaro’s government (2019 to 2021).^2^ Analyzing the case of Brazil also allows us to provide an important contribution to the literature: the country is characterized by high levels of political polarization, and the quality of democracy has decreased since 2016 (Coppedge et al. 2023). Brazil’s political landscape is particularly worrisome given the historical success of authoritarian leaders as well as its media environment being one of high clientelism and commercialism (Paiva, Sodré, and Custódio 2015). Finally, Brazil is the largest Latin American country in terms of economy, territory, and population, and is therefore worthy of explanation in its own right.

In the next section, we review the democratic and social role of the news media, which creates a dilemma regarding what should be published or not. We then refer to some insightful quantitative studies that have analyzed what has been published by legacy news outlets and detail why the absence of qualitative work focusing on journalists’ norms and practices is an important gap that needs to be filled. Our use of semistructured in-depth interviews is set out in detail in the methodology section before the presentation of our findings. Finally, we conclude by discussing the implications of our results for understanding the interaction between the media and politics.

## Journalists’ Practices and the Role of Their Perceived Impact on Public Opinion

Several studies offer evidence that news media can enable and facilitate a state’s progress throughout the stages of democratization (McConnell and Becker 2002; Voltmer and Rawnsley 2019). A significant strand of this scholarship also argues that legacy journalism can sustain and protect democracy after a regime transition is consolidated (McQuail 2000; Street 2001). The democratic roles of the press include the fair mediation of information and representation of political events guided by the public interest (Schudson 2008; McNair 2009). Specifically, research on political transitions demonstrates that journalists and news organizations shape democratization by enabling public deliberation and holding those in power to account (Randall 1998; Schedler 1998). As argued by Mughan and Gunther (2012, p. 1), the news media is “the connective tissue of democracy” because it is “the principal means through which citizens and their elected representatives communicate in their reciprocal efforts to inform and influence.” Moreover, the continual transparency and accountability provided by quality news reporting improve the quality of elections, branches of the state, and civil society over time (Daoust and Nadeau 2023; Norris 2006; Schudson 2008). Consequently, the democratic function of news media encompasses the production of news outputs that support citizens’ informed decisions and reinforce democratic attitudes.

Some kinds of news outputs, however, can endanger the news media’s prodemocratic role by negatively affecting public opinion. Previous studies of media effects show that increased visibility of certain voices, perspectives, or incidents can enable violent and antidemocratic attitudes (Blassnig et al. 2019; Brown and Mondon 2021; Donovan and Boyd 2021). For example, news reports on the lynching of Black people are thought to have normalized white supremacist crimes in the United States, which undermined reform and accountability in the 1950s (Wood 2009; Berger 2011). Recently, Brown and Mondon (2021) showed that legacy news media in Europe contributed to the mainstreaming of radical far-right political actors by both “trivializing” and “amplifying” their discourse over the past decade. Similarly, Blassnig et al. (2019) showed that populist messages in news articles not only generate more comments from the readership but also prompt citizens to utilize populist messages in their own comments, including antidemocratic discourse. Studies also suggest that news outlets in the United States politicized the COVID-19 pandemic by giving more space to political actors than to health experts, which may have contributed to polarization and riskier health attitudes among citizens (Hart, Chinn, and Soroka 2020). In Brazil, previous studies suggest that Jair Bolsonaro’s discourse increased polarization on sociodemographic cleavages, such as race and gender (Layton et al. 2021).

In this context, there is a dilemma among journalists regarding the publication of news content, as this can have a negative effect on public opinion vis-à-vis their responsibility to provide information for public deliberation and hold social actors to account. Numerous studies have analyzed this dilemma through the framework of gatekeeping (Shoemaker and Vos 2009) to explain news selection (Jacobs and van Spanje 2023) and examine journalists’ attitudes (Kim 2010). Gatekeeping theory conceptualizes reporters and editors—and their news organizations more broadly—as gatekeepers who close or open the gates, thereby constraining or facilitating the flow of information (Shoemaker, Vos, and Reese 2009; Shoemaker and Vos 2014). In other words, many events occur, and countless amounts of information are available, from which journalists as gatekeepers need to make a selection.

However, this gatekeeping process is mostly structured around the notions of “news values” and “public interest” to explain why some events or issues are more “newsworthy” than others (O’Neill and Harcup 2009; Harcup and O’Neill 2017) rather than concerns with public opinion and well-being. The activities of the political elite are widely accepted as newsworthy or even essential to public deliberation and democracy (Schudson 1995; McNair 2009). Still, little is known about journalists’ attitudes when information that is seen as essential for democracy might also be harmful to the public, for example, by spreading misinformation or fueling hate and threat of (political) violence (Ferreira and Alcantara 2023).

Gatekeeping theory has also been widely leveraged to study journalistic routines, organizational pressures, social-institutional factors, and social systems of influences (Shoemaker and Vos 2009). However, theorizing on the mechanisms through which these influences manifest is scarce (Bro and Wallberg 2015; Tandoc 2018), and we know little about the rationale or the “inner-conversation” of journalists in such instances (Wright 2011). Moreover, the framework implies that journalists are always susceptible to influence, when, in practice, journalists are also capable of resisting such pressure (Tandoc 2014).

Hence, there is an important gap in the literature regarding qualitative analyses designed to explore the decision-making process in newsrooms. Scholars investigating news selection tend to use more content analysis (Shoemaker et al. 2001; Jacobs and van Spanje 2023) or large surveys (Kim 2010; Ghersetti and Johansson 2021) than in-depth qualitative explorations of journalists’ perceptions and decision-making (Reich and Barnoy 2016; Hoxha and Hanitzsch 2018). Yet, content analysis cannot investigate what was not published or the rationales of journalists involved in the decision—indeed, data regarding what was not published are often not publicly accessible.

Political science studies specifically concerned with the effects on public opinion are, to some extent, limited to quantitative content and text analyses. Among others, Brown and Mondon (2021) and Hart, Chinn, and Soroka (2020) conducted a content analysis of news articles in order to identify dynamics of normalization and amplification of harmful content or antidemocratic discourse. Similarly, Sbaraini Fontes and Marques (2023) analyzed social media content produced by newspapers to demonstrate how they amplify the visibility of illiberal populist leaders. Blassnig et al. (2019) demonstrated such amplification by conducting a content analysis of populist messages in online news articles and corresponding reader comments. However, the perceptions and rationales of journalists regarding the publication of such content are considerably understudied.

Qualitative interviews with journalists can provide a valuable understanding of the mechanisms of influence in news selection and journalists’ rationale, as well as the implications for their democratic role. News production is a process negotiated between a number of different professionals (Raemy, Hellmueller, and Vos 2023), balancing different commitments as well as formal and informal rules (Deuze 2007; O’Neill and Harcup 2009). Individuals and groups make decisions by “weighing” different and (often conflicting) systems of meaning, attribution, commitments, and obligations (Archer 2000). Our research will, therefore, contribute to knowledge by providing a new perspective through a qualitative approach that analyzes journalists’ rationales regarding the publication of content they perceive as potentially harmful to public opinion.

## Research Design

### Case Study

These qualitative data were collected as part of a larger project that investigates the role of mainstream news organizations in key political events in Brazil between 2016 and 2021 (Ferreira 2024a). Brazil is a useful case study for our research question, as it expands our understanding of the role of journalists’ perceptions of public opinion, norms, and practices. Brazil is important for many reasons. (1) It is the largest country (both demographically and economically) in Latin America, but one that is understudied compared to many other countries. (2) Brazil is the fourth largest democracy in the world. (3) The country’s sociopolitical landscape is particularly pertinent for researching media and politics.

Among other things, Brazil has an important history of authoritarianism (the military regime ended in 1985), which reinforces the need to understand the democratic role of the news media. Moreover, the quality of Brazilian democracy has decreased between 2016 and 2021, according to the Varieties of Democracy Project (Coppedge et al. 2023).^3^ This is particularly worrisome given that it has been accompanied by the success of authoritarian leaders who often use the media environment to their advantage (Hunter and Power 2019). Content analysis of news outputs suggests that Brazilian legacy news organizations provided unfair representations and unbalanced coverage during this period (van Dijk 2017; de Albuquerque 2019; Araújo and Prior 2021). Specifically, they appear to have facilitated Brazil’s de-democratization by targeting specific political actors and favoring commercial interests (Ferreira 2024b).

### Methodological Approach

In this research, we seek to provide a fresh perspective on journalists’ perceptions of public opinion, norms, and practices by leveraging qualitative data. More precisely, we conducted semistructured in-depth interviews, which can be an effective method for tapping into elites’ public opinion and exploring journalists’ practices, rationales, and perspectives (Plesner 2009; Karlsen and Stavelin 2014; Ferrucci 2017). The semistructured form combines open questions with strategic follow-ups for clarification (Wengraf 2001). Hence, this method provides a balance between granting participants the relative freedom to talk about specific situations and returning to more rigorous questioning when the conversation slips to shared “common-sense” assumptions (Brinkmann and Kvale 2014).

We focused on the mainstream news organizations with the largest audiences, as determined by the Reuters Digital News Report, which remain highly influential for public opinion in Brazil (Newman et al. 2021, pp. 116–17). These are (1) Globo Group, which includes the country’s leading TV network, a 24-hour news channel, and news websites; (2) Record Group, which has similar outlets and ranks second in TV audience; and (3) Folha Group, which controls the most influential newspaper and the biggest news portal of Brazil. As such, the choice of media groups is based on “strategic sampling” (Mason 2018, p. 58), which is designed to capture a relevant range of a wider population but not to be representative per se.^4^ However, our interview sample was designed to broadly represent the production chain of political news in these organizations, including both journalists responsible for covering political events (e.g., reporters and producers) and those in the decision-making process of publication (e.g., editors and media managers).

The participants were selected based on open-ended desk research (Bastian 2019, p. 195). Journalists covering politics or managing newsrooms for Globo, Record, and Folha were identified and selected through the news coverage of selected political events. For this study, temporally, we focused on the 2018 presidential election campaign (January 1 to October 31, 2018) and the first three years of Jair Bolsonaro’s government (January 1, 2019, to December 31, 2021). Bolsonaro was the first far-right politician to become president since the country’s democratization in 1988, and the first to challenge key democratic norms both as a major candidate and as a sitting president (Hunter and Power 2019).

These news outputs were retrieved using the news organizations’ digital archives on their websites and streaming apps. Then, we used byline information from the news outputs to assemble a list of 67 potential participants. Subsequently, interviewees recommended another 14 journalists involved in the news coverage, which allowed us to identify professionals not credited in the bylines (e.g., producers and assistant editors). This snowballing technique does not threaten the external validity of the sample.^5^ On the contrary, this strategy increases the likelihood of a more balanced representation of the news production chain in the chosen media groups because it allowed us to find relevant names that were not publicly disclosed in the news article.

The news coverage of both the 2018 election and Bolsonaro’s government includes numerous articles from 2018 to 2021. All reporters and producers identified in our open-desk research and through snowball techniques covered both cases multiple times, which is to be expected as these were key political events typically assigned to the leading members of the political team—a relatively small and stable group. Editors and assistant editors in the selected Brazilian news organizations usually oversee all outputs in their “news beat”; thereby, selecting those working in this time period automatically included those engaging with both cases. Moreover, during this period, all three organizations had the same media managers and high-level editors, which is also to be expected given the slow turnover in senior positions. As a result, all potential participants in our initial target list, by the design of our selection process, were journalists deeply involved in the news coverage of our two cases, allowing us to explore specific production processes and decision-making, rather than just general opinions.

The initial list and the recommendations made up a target of 81 journalists. A total of 36 agreed to participate and were interviewed—10 in person (in Brazil) and 26 online (i.e., video call systems, such as WhatsApp and Skype). This is an acceptance rate of 44.4 percent. However, many interviewees had to be contacted several times and talked into participating through different channels (i.e., email, cellphone, and social media like WhatsApp). Potential participants presented two key concerns: lack of time for the interview and fear that their identity would be uncovered. To maximize the odds that they agree to participating, we explained our commitment to research ethics and preserving their confidentiality. We also emphasized that they would contribute to knowledge production and improve our understanding of newsmaking, potentially leading to a better understanding of journalistic practices. Thirteen journalists still declined an interview, and 22 did not reply to our messages. Another 10 journalists initially agreed but stopped replying to our requests to set a date for the interview.

We also consider the news organization and the professional position of the participants when pursuing the interview requests. Specifically, we maintained a balance across the three media groups (see table 1) and aimed for a proportion of professional positions that is as representative as possible of the production chain found in Brazilian mainstream newsrooms (see table 2)—around 50 percent of reporters, 30 percent of editors, and 20 percent managers (Bastian 2019). Regarding the individual characteristics of the interviewees, 17 participants had between 10 and 20 years of experience, 16 were in the range of 21 and 40 years of career, and three had more than 40 years of journalistic experience.

**Table 1.** Participants per news organization.

News organization	Description	Participants (N = 36)
Globo Group	More than half of these participants worked for TV Globo. Founded in 1965, TV Globo is the oldest nationwide television network operating in the country and has been the leading channel for over half a century. Around one-third of participants worked for the group’s website (G1) or their all-news cable channel (GloboNews).	11
Record Group	More than half of these participants are from Record TV, the second most-watched TV network, or the group’s all-news cable channel (RecordNews). Almost half worked for their website (R7).	10
Folha Group	The Folha Group controls the newspaper Folha de S. Paulo and the online portal UOL. Founded in 1921, Folha is considered one of the most influential newspapers in Brazilian politics. It has the largest national distribution and is the number one for printed and digital subscriptions. UOL is the most accessed in the country and rivals G1 in news audience. Number of participants is similar for each outlet.	15

**Table 2.** Participants per professional position.

Professional position	Participants (N = 36)
Profile 1: Reporters and TV producers	21
Profile 2: Editors, assistant editors, or similar job details	12
Profile 3: Media managers (executive positions such as editor-executive, editor-in-chief, or newsroom director)	3

Further detail on the recruitment process is provided in Supplementary Material table S1. We also compared the profile of the achieved sample and those who were not interviewed, which contained no significant variation (see Supplementary Material table S2).

### Conducting Interviews

The in-depth interviews were conducted between December 7, 2021, and July 20, 2022, and lasted, on average, one hour and a half. The research was conducted with the ethical approval of the University of Edinburgh. All participants were given the right to have their identity remain confidential. This measure was adopted to protect their careers and personal safety since they were expected to provide detailed information on their working practices and on political issues in a context of increased political tension in Brazil, which would then be made publicly available in an academic publication.

The interview questions (see Supplementary Text: Interview Questions, pp. 2–5 in the Supplementary Material) were designed to explore how journalists covered the selected political events (i.e., the 2018 elections and Bolsonaro’s government), their rationale when doing so, and the chain of decisions within the newsroom for the resulting news outputs. Therefore, several questions resulted in valuable data on the journalists’ decisions about what kind of content should be published or not.

Interview questions were kept strategically broad to avoid influencing the answers and increase the validity of the data. Instead of directly questioning what was published or not, and what may be nonpublishable (or problematic) content, the interview script included questions such as “How did you (or your colleagues) decide to tell these stories?”, “What conversations did people have during the editorial meetings”, and “How did you decide what to pursue or not? What to highlight or not?”

In addition to the questions that were directly relevant for our inquiry, strategic follow-up questions adjusted in real time were particularly useful in getting more details on the journalists’ rationales for publication and overcoming post-hoc rationalization of answers. These were employed in three main situations: (1) When the answer was not substantial enough, and further clarification was needed. (2) To overcome or clarify what was perceived by the interviewer as rationalization or “shared common sense.” (3) When the answer presented significant discrepancies in relation to one or more of the following: (a) related news coverage (which was previously reviewed by the interviewer before the interviews); (b) previous studies with content analysis of related news coverage (van Dijk 2017; Araújo and Prior 2021); and (c) the accounts of other participants from the same news organization. As such, participants’ accounts of their actions were thoroughly investigated before being subjected to analysis.

Some participants often started with rationales around traditional ideals of impartiality, balance, and protection of democracy. The degree of similarity was enough for the statements to sound rehearsed. Building on the techniques of previous studies on decisionchains in journalism (Reich and Barnoy 2016, 2020; Hoxha and Hanitzsch 2018), we developed a subtle but firm approach to move away from abstract statements. This process included variations of the replies “I wonder how the conversation was in your team” and “But, looking at these specific news outputs, it seems to have gone in a somewhat different direction.” More rigorous follow-ups were then employed using news outputs from the period and previous studies. For example, one journalist said that election coverage should avoid “candidates attacking each other and focus on policies,” but we followed with “However, your newspaper published these kinds of attacks several times” and cited some news items. In another recurrent rigorous probing, generic claims that the “politics newscast is always balanced” were confronted with studies suggesting that the press downplayed Bolsonaro’s authoritarian values or applied different standards for critical approaches, which disproportionately benefited him and/or undermined his rivals.

While the procedures discussed so far were largely effective, it is important to note some limitations in our data collection approaches. The data collected were dependent on the participant’s ability to remember events that had occurred relatively far back in the past (Anderson and Jack 2016). Interviewees in qualitative studies can also be purposefully generic, evasive, and/or engaging in post-hoc rationalizations (Mason 2018, p. 254). The follow-up questions were used efficiently to minimize such effects, as they maintained a focus on specific practices employed in the news coverage of each case study. Securing multiple participants from the same news organizations also allowed us to triangulate accounts and flag significant inconsistencies in their reports (Rubin and Rubin 2005, p. 136). Moreover, the follow-ups that were informed by additional documentation allowed us to overcome evasiveness and rationalization, improving the quality of the accounts provided.

All interviews were conducted, transcribed, and translated by one of the authors, who is also a native Brazilian Portuguese speaker and a former journalist. This allowed us to ensure careful consideration of the aims and scope, as well as maintain the criteria and strategies described in this section throughout the data collection process.

### Active Reflexivity

The professional and cultural background of the interviewer (i.e., a former Brazilian journalist) offered more access to interviewees and greater knowledge of the diverse journalistic operations in the country. It became clear in several interviews that the “insider status” (Couture, Zaidi, and Maticka-Tyndale 2012) of the interviewer made participants more comfortable talking in detail about their practices and the internal dynamics of their news organizations, which is usually sensitive to journalists. After a long pause, the expressions “Okay, I will tell [you] because it is you” or even “because I know you will keep my name off it” were common. These interviewees saw the interviewer of this study as a more “sympathetic” listener and more reliable regarding confidentiality due to his previous experience as a journalist in Brazil. Building on previous approaches (Bowd 2004), the interviewer used his “insider status” to create a more comfortable environment when discussing challenging topics, leading questions with “I remember from my time as a journalist.” Moreover, the interviewer’s background makes him a skilled listener in qualitative research overall, even more so when participants are speaking in his native language about his home country.

By contrast, the “insider status” of the interviewer also meant that he was subjectively close to the participants because of their shared professional experience and cultural background (Ganga and Scott 2006; Zempi 2016). However, this condition, it has been argued, can threaten the researcher’s ability to provide objective knowledge (Wilkinson and Kitzinger 2013; Collins and McNulty 2020). A significant advantage of “outsider” researchers is the ostensible objectivity that being detached provides, which enables them to find meanings not evident to the insiders (Savvides et al. 2014). In practical terms, a former journalist risks being “too soft” with former colleagues or a “disproportionate critic” due to past grudges toward the news industry. These perceived challenges to objectivity during data collection and analysis would then have the potential to threaten the validity of research outputs.

Therefore, to reduce the potential risks, we adopted a posture of “active reflexivity” (Fujii 2018; Soedirgo and Glas 2020) in every step of the study. This includes the author who was the interviewer during data collection, and both authors throughout the analysis. According to Soedirgo and Glas (2020, p. 530), being actively reflexive means “engaging in the dynamic, continual, and fluid practice of interrogating our own assumptions of positionality, how positionality is being read by others, and the impact of these assessments throughout the research process.”^6^ More than a habit or a set of procedures, this is “an embodied disposition toward reflexivity as research is conducted” (2020, p. 527). As such, active reflexivity is applied and observed in every step—research design, data collection, and analysis.

In addition to this broader posture, we implemented specific procedures based on reflexivity, adapting protocols designed by previous qualitative studies (Engward and Davis 2015; Soedirgo and Glas 2020; Olmos-Vega et al. 2022). This entailed frequently producing notes and mind maps of decision-making during interviews and analysis, followed by further reflection with a fresh mind before reaching a final decision. As such, we anticipated multiple perspectives of criticism and avoided echo chambers, weighing a wide range of sources when conducting the analysis (Fujii 2018). These sources included academic literature, empirical data, op-eds by media experts, and news articles for additional context, depending on the decision to be made. These notes and reflections throughout the project allowed us to constantly question perceptions of bias and improve our actions accordingly.

The written materials of our reflexivity process cannot be disclosed, both to protect against the identification of the participants and due to its largely informal format. However, key research developments and interrogations were shared with two colleagues not involved in the project, who are world-class academics in this field from countries other than Brazil, one of whom is not a former journalist. This practice follows previous approaches to mitigate the limits of self-reflexivity that recommend bringing other scholars into your reflections, thereby sharing your interrogations, decisions, and research developments with those who can shed light on dynamics that may have escaped you (Soedirgo and Glas 2020, p. 530). Finally, we rigorously followed our research design, which was developed to meet the high ethical standards required by the University of Edinburgh.

### Analyzing the Data

After transcribing the participants’ accounts, we analyzed all interview data through open textual coding using NVivo. We chose NVivo due to the length of the interview transcripts and the need to analyze the data multiple times within the larger project this study is part of. Although we considered alternative software, NVivo was the most suitable option based on the team’s prior experience and its effectiveness for data management and analysis in similar studies (Bazeley and Jackson 2013; Jackson 2019). This open textual coding on NVivo allowed us to systematically organize, categorize, and annotate key phrases and segments that related to our research questions. The purpose of this approach was to identify recurring patterns and insights from the data to provide a grounded understanding of journalists’ perceptions of potentially harmful content and their rationale(s) for publishing or withholding it.

In doing so, we conducted a thematic analysis, to systematically identify, organize, and interpret patterns (themes) within the interview data (Braun and Clarke 2006; Harper and Thompson 2011; Braun et al. 2018). Previous studies show that this approach is highly effective in identifying and interpreting patterns, particularly within qualitative data (Miles and Huberman 1994; Maxwell 2004; Braun, Clarke, and Weate 2016). A thematic analysis starts with coding, labeling key data segments with short descriptors (e.g., “family” or “time”) (Clarke and Braun 2017). These codes are grouped into broader themes, which summarize recurring patterns or ideas (Braun and Clarke 2012). However, Braun and Clarke 2019) emphasize the researcher’s role in actively shaping themes, not merely “discovering” them. While “discovering” implies that themes exist inherently within the data, waiting to be uncovered, “shaping” suggests that researchers play an active role in interpreting and constructing themes based on their analytic lens and research context. Overall, thematic analysis helps organize data into meaningful patterns, making it suitable for exploring qualitative research questions (Braun and Clarke 2023).

For developing codes, thematic analysis allows for both deductive (theory-driven) and inductive (data-driven) strategies. Following Gilgun (2019), Mayring (2000), and Saldaña (2021), we employed a deductive-inductive process in which codes were constructed based on both existing theory and the gathered data. We started by carefully reading the interview transcripts and identifying key segments of the text that aligned with the research questions. Codes were assigned to segments of text to label specific ideas, such as “misinformation” for the journalists’ descriptions of Bolsonaro’s statements they considered misleading or “authoritarian values” for those, for example, advocating the closure of Parliament or attacking of the press. While codes such as “misinformation” were in our initial list because previous studies argued that misleading information is a point of contention in political news coverage (Ferreira 2022), we also developed codes based on the recurrent patterns in the data. For example, several journalists talked about the need to explain or correct misleading statements (i.e., coded as “inclusion of additional context”) or to publish such statements only when a new one is made, rather than amplifying repeated misleading narratives of political actors (“reducing coverage”).

Codes were then refined through multiple rounds of coding, with some codes being merged or eliminated as we worked toward a final codebook. For example, early in the coding process, we coded the participants’ argument that news coverage should give voice to multiple political voices in slightly different forms (e.g., impartiality, balance, fair competition). However, these sections of the transcript can all be identified under the central idea of “pluralism.” Likewise, journalists’ accounts regarding the inclusion of additional context or additional explanations when publishing disinformation were initially coded separately, but later merged as the same code due to their evident similarity.

The next phase involved grouping the coded data into broader themes. A theme represents a broader pattern in the data, capturing essential aspects related to the research question. For instance, the theme “perceptions of problematic content” grouped together codes like “misinformation” and “authoritarian values,” while references to “pluralism” always appeared as a rationale for publication (“reasons to publish”). Similarly, “inclusion of additional context” or “reduced reporting” were grouped under the theme “mitigating measures,” as these accounts consistently referred to the journalists’ intention to publish problematic content. Therefore, we discussed and analyzed the similarity and context of codes in relation to our research to create themes or thematic axes as broad organizing principles to which codes were then allocated.

Subsequently, we analyzed how different codes interacted within these thematic axes. For example, the code “hate speech” was often associated with journalists’ decisions not to publish certain content. This relationship suggested that journalists perceived the amplification of hate speech as a violation of professional standards, thus providing a rationale for “not to publish” (another theme). Another example is how journalists cited the need for “accountability/denounce” as a reason to publish controversial statements, even if they contained “misinformation.” Table 3 shows the final list of themes and codes.

**Table 3.** List of themes and codes.

Themes	Codes
Perceptions and definitions of “problematic” content	Misinformation Authoritarian (or antidemocratic) values Hate speech
Reasons to publish	Pluralism Accountability/denounce Legitimacy of the far-right (Bolsonaro) Audience concerns
Reasons not to publish	Failure of social and democratic functions Conflict with professional standards or codes of conduct
Mitigating measures	Inclusion of additional context or explanations Reduced reporting (only new statements/topics)

This process of organizing and coding the data allowed us to develop meaningful patterns and insights, which informed the discussion of our findings.^7^ In the next sections, we present these findings, structured around these four key themes: (1) perceptions and definitions of “problematic” content, (2) reasons to publish, (3) reasons not to publish, and (4) mitigating measures.

## Findings

Our thematic analysis of the interview data allowed us to identify four main trends. The first theme comprises the journalists’ perceptions and definitions of “problematic” content. Specifically, participants reported a growing concern with particular kinds of statements or conduct by political actors that could misinform, increase polarization in public opinion, and even spark violence. The second theme comprises the journalists’ decision-making process and rationale for publishing this content. A third group of answers describes the decision-making process and rationale for *not* publishing. Finally, a fourth theme aggregates the measures adopted by journalists in order to mitigate harm to public opinion when “problematic” content is published. Their perceptions regarding the effectiveness of such measures were also included in this category for analysis. We analyze these four key findings in detail below.

### Perceptions and Definitions of “Problematic” Content

The majority of the participants raised concerns regarding statements made by Bolsonaro and some of his close allies. These statements were made during the 2018 election campaign. The in-depth interviews also revealed an increase in such concerns after Bolsonaro was elected. Most of these accounts also reflected political tensions with an escalation of antidemocratic statements by a sitting president or key government figures. Moreover, this escalation frustrated the expectations of some journalists that Bolsonaro would tone down his criticism of democratic norms once in office. According to the interview data, three key types of content were perceived as “problematic” by journalists in the analyzed period. These are statements considered as potentially harmful to public opinion, which generated inner/individual or collective discussions about their publication.

#### Misinformation

The first type refers to statements containing what was considered *misinformation* by the journalists. Most examples provided by participants related to environmental issues and the COVID-19 pandemic. Bolsonaro often provided misleading information on fires and deforestation in the Amazon, including what interviewees described as “numbers mathematically impossible to be true.” The president and his government also denied or downplayed the risks of the pandemic, discredited vaccination, and incentivized citizens to not wear personal protective equipment, such as masks. What journalists found particularly problematic was those statements that contradicted scientific evidence and the views of the relevant health authorities, including his advocating for unproven treatments, as well as his public appearances without a mask and rallies that violated social distancing rules. As reported by nearly all participants, the rationale for classifying this type as “problematic” is that they risked undermining the formation of well-informed public opinion regarding crucial issues and, thereby, enabling inadequate or risky behavior among citizens.

#### Authoritarian (or antidemocratic) values

The second type perceived by journalists to be problematic includes content with *authoritarian (or antidemocratic) values*, which was also raised by most interviewees. These were described as Bolsonaro’s attacks or “inadequate conflicts” with the opposition and other institutions, such as the Parliament and the Supreme Court. During an election campaign rally in September 2018, Bolsonaro said “let’s shoot the *petralhada* [supporters of the opposition^8^] or send them to Venezuela” while wielding a camera tripod as if it were a machine gun (Bolsonaro 2018). Moreover, as president, he advocated closing the Supreme Court and suggested eliminating other checks and balances. Interviewees also reported Bolsonaro’s “violent discourse” against the press in general or toward specific journalists. The president also often praised several authoritarian regimes, their leaders, and some known torturers, including the Brazilian military dictatorship (1964–1985). According to participants, these kinds of statements were potentially harmful because they could further polarize public opinion and even increase support for authoritarian values. Moreover, they led to Bolsonaro’s supporters physically attacking journalists, which undermined their work and their access to information.

#### Hate speech

Finally, a third group of statements made by Bolsonaro and some of his allies were considered by interviewees as a form of *hate speech*. These were racist, misogynistic, homophobic, or transphobic remarks. For example, Bolsonaro said that “staying at home is for sissies” and wearing masks was “a fag thing” during the COVID-19 pandemic (Ferreira and Alcantara 2023). In July 2022, the president said that “John will be John, and Mary will be Mary for life” and that his model of family is “Man, Woman and offspring” (Portilho 2022). Additionally, the president often dehumanized Indigenous and Black peoples (Teixeira 2022). Moreover, his remarks depicting women as submissive or less important than men are well known, including saying to a member of the Parliament that “she does not deserve to be raped because she is too ugly” (Ramalho 2016). Among many participants, there were significant concerns that publishing such statements would legitimize prejudice and even risk increasing physical violence toward people of color, the LGBTQIA+ community, and women. In 2022, there was a 67 percent increase in reported hate crimes in the country compared to the previous year (Cruz 2023).

### Reasons to Publish

The previous section detailed what kinds of content were considered problematic. In other words, it described the conditions under which journalists are faced with this dilemma. We now move to the reasons *why* journalists decided to publish some content fitting this description. Within this category, we found much less consensus than on what constituted problematic content. The participants’ accounts indicate a wide range of arguments with the key themes being mentioned by many of them. Nonetheless, some important trends can be found. (1) They mentioned that journalists should report on “all competing voices in politics,” which would include candidates in the election, members of an elected government, and the opposition. (2) Concerning the accountability role of the press, the interviewees raised the idea that potentially harmful statements should be published as a form of denunciation, particularly after Bolsonaro became president. (3) Journalists observed that the 2018 election period created a more specific dynamic in which the rationalization of Bolsonaro as “a legitimate candidate” connected with the political interests of some news organizations. (4) A few participants reported that not publishing “problematic” content related to Bolsonaro would lead people to believe that they are “left-leaning.” Finally, these last two arguments were also presented in connection with broader audience concerns, as Bolsonaro’s statements attracted high engagement on social media. We will discuss these rationales in detail.

A recurrent rationale was that “reporting all competing voices,” including problematic ones, is part of the journalists’ role in informing the public, even if the discourse contains misinformation or hate speech. Interviewees also said this content should be published precisely for the purpose of pointing out its inadequacy, that is, as an accountability mechanism or “to denounce [it] as wrong.” Interview data also show that these forms of rationalization increased after Bolsonaro became president. For example, participants said:

We need to report what the president is saying. You cannot ignore when the president says something… even if it is to show that it is absurd or to denounce it. (Journalist 16—Profile 2)

The President of the Republic is the highest authority in the country, right? If you don't show what he says, you are giving up on historical record-keeping and abandoning your journalistic duty. (Journalist 6—Profile 2)

Many participants justified publishing Bolsonaro’s polemic statements during the election campaign because he was “a legitimate candidate,” who was running “within the democratic process” (e.g., Journalist 30). Among others, this senior editor supported this rationale:

We were criticized for giving space to Bolsonaro at the beginning of the electoral campaign. Oh, you are normalizing Bolsonaro. And our response was, “he is a candidate.” The democratic system is normalizing him. It accepted him and he is a candidate. (Journalist 6—Profile 2)

However, many interviewees also reported an overrepresentation of Bolsonaro during the election campaign following requests of media managers and high-level editors. Underlying these requests were an open preference for Bolsonaro at Record Group and the strategic support for Bolsonaro or “partisan interpretations of balance” at Globo Group and Folha Group, as identified by previous studies (Ferreira 2024b). As a result, decisions in news production that amplified statements with misinformation and hate speech were normalized. As explained by this participant:

If you are going to publish a news item about PT, you have an obligation to make one about Bolsonaro. You have a good news item that involves left-wing topics or actors. Then you will have to dig up some Bolsonaro shit to put it on the side. That is when you end up highlighting things that shouldn’t be highlighted … reverberating populism and hate. (Journalist 22—Profile 2)

Some of these participants also reported that a general fear of being seen as left leaning has led to the publication of such statements. Reporters feared being branded as left leaning by superiors in news organizations, themselves owned by business elites who are traditionally unenthusiastic toward left-wing leaders. One participant explained how this dynamic became more relevant in a period of growing polarization between Lula and Bolsonaro:

The newspaper often slips into childish criticism of Lula and is terrified of his party. The direction operates with the premise that journalists are more left-leaning and they have to hold back. Anyone who works at the newspaper is terrified of hearing that they are petista [Lula’s party supporter] or left-leaning. You don’t want to have that blemish. (Journalist 11—Profile 2)

The fear of being seen as left leaning was more prevalent at the organizational level during the period of study. Specifically, high-level editors and managers feared alienating part of their audience in the context of growing support for Bolsonaro. Many participants confirmed that audience concerns were often factored into the decisions regarding the publication of statements that were already considered misinforming or authoritarian by both the candidate and the president Bolsonaro. As this participant described:

Of course, audience, profits and financial pressure were also part of the discussion. We have instant audience metrics and pressure. And every time you put Bolsonaro up in a headline, you get more audience on the website. It just explodes. You attract people supporting and people criticizing. (Journalist 1—Profile 1)

Our results in this theme further corroborate Panievsky’s (2022) concept of “strategic bias,” according to which journalists might lean toward a particular position and provide unfair representations to avoid backlash from the public. However, the case of Brazil shows two key differences: (1) The role of media managers and high-level editors seems more pronounced and systematic. (2) Strategic bias can affect more than the fairness of news reporting and enable content potentially harmful to public opinion.

### Reasons Not to Publish

Many of the journalists provide accounts of occasions in which there was discussion that could lead to not publishing the contents perceived as harmful to public opinion. The rationale provided in these accounts was articulated within two central themes. Journalists perceived the publication as a failure of their social and democratic functions or a contradiction of their professional standards or codes of conduct. Overall, there was a general sentiment among the interviewees that the publication of these statements was something they “shouldn’t do as journalists.”

These two central ideas were described with variations depending on the different types of content. For example, publishing statements with misinformation would undermine their role in informing the citizenry. Similarly, amplifying messages that promote division or authoritarian values would be a failure for journalists perceiving the protection of democracy as part of their social functions because they might erode democratic attitudes. Finally, publishing statements that could be perceived as hate speech was articulated by the participants as falling below standards or even as a violation of professional codes. Representative of the interviewees echoing these views, one participant explained how they strongly advocate against giving space for political actors who were providing such statements:

You do not give space in the debate for flat Earth activists to provide balance. Don’t we evolve a little as a society? Some consolidated issues, such as the scientific process, democracy, and human rights, do not require balance. Otherwise, you risk amplifying lies and legitimizing something that you know is not true or even outright prejudice. (Journalist 5—Profile 1)

The interview data also suggest that a divergence in the rationales discussed above is contingent on the journalist’s professional positions and status in the newsroom. Specifically, the concerns and formal manifestations against publication during the news production process were significantly higher among reporters and producers, who are at the lower levels of the newsroom. High-level editors and media managers demonstrated less concern with publication and were more frequently associated with the pro-publication rationales presented in the previous section. This trend reinforces the validity of previously discussed reports on political preferences and partisan interpretations of balance that originate from top positions that shape news outputs.

Finally, the concerns and rationales against the publication of potentially harmful content were reported by many participants as “being raised” in the negotiation among professionals that characterizes news production. However, most interviewees acknowledged that pro-publication arguments frequently won in these negotiations,^9^ even with the addition of mitigating measures to these publications. For example, this seasoned journalist suggested that a key pro-publication argument (audience concerns) was dominant between 2016 and 2021:

Without a doubt, a social wave influenced journalistic coverage at that time. It was a period when social media was on the rise, with strong polarization. People on social media wanted polemics, accusations, attacks … not balanced and contextualized information. Journalistic production aimed to meet this demand precisely to ensure an audience. The wave, right? You go along with the wave as it carries you. This shaped most of the decisions. (Journalist 1—Profile 1)

### Mitigating Measures and Conflicting Perceptions of Effectiveness

Another recurrent theme in the interview data is the mitigating measures. Many interviewees reported the use of specific practices to justify the publication of statements previously considered harmful to public opinion.

The practice most described by interviewees was the inclusion of additional context or explanations to minimize potential negative impacts. This is explained by the following participant concerning Bolsonaro’s views on the pandemic:

We started to be more careful and make sure every misleading or inflammatory statement was companied by the correct data or a reputable expert that would correct or at least show a different, critical perspective. (Journalist 16—Profile 2)

In addition to the contextualization, a few TV editors also decided not to broadcast certain statements. This journalist described a form of “indirect broadcast” adopted by “Jornal Nacional,” a primetime news segment from Globo:

We made a decision during the pandemic to significantly restrict Bolsonaro’s nonsense from being broadcasted live. So, we rarely showed a scene of him with his own voice, saying something absurd. This was done indirectly. It’s the news presenter saying, “in a live broadcast on social media, the President said that COVID-19 vaccines cause AIDS, which is not true,” instead of showing the scene of Bolsonaro speaking. (Journalist 24—Profile 1)

The second most recurrent mitigating measure among these participants was to only report a harmful statement if it was completely new. According to the interviewees, Bolsonaro and his allies often tend to repeat key lines of discourse. As such, the editors of some of the news outlets in our study decided to implement the rule of publishing harmful statements only if said for the first time. In relation to Bolsonaro’s presidency, this editor said:

One of the things is to know if what he said wasn’t already a lie he has told before. If he keeps repeating the same spiel, we won't publish it again. But like it or not, he is the President of the Republic. It's inevitable. He is someone whose statements you need to publicize. Even to question them. (Journalist 4—Profile 2)

Finally, toward the end of the analyzed period, a ban was introduced by media managers in some news outlets, and journalists stopped covering Bolsonaro’s daily talks with the press. However, the participants’ accounts suggest that the decision related more to concerns for journalists’ safety rather than the need to avoid damaging changes in public opinion. That is, the decision came after the president’s supporters physically assaulted journalists following an escalation of his anti-press statements. Nevertheless, these outlets continued to report some of Bolsonaro’s speeches using social media lives and videos.

However, nearly all participants were highly skeptical of the effectiveness of these measures and how (in)consistently and (un)fairly they were employed. Among others, this participant expressed their disbelief in the capacity of mitigating measures to protect the public or stimulate their critical engagement:

I doubt of the real effect. We report some of his statements to highlight how inadequate it is. But a large part of the audience will just read Bolsonaro said climate change is lefty-wing bullshit, and he is right. (Journalist 5—Profile 1)

Overall, the participants’ reports suggest that adopting some mitigating measures led to a rationalization of the publication of harmful statements. However, the accounts within this theme also indicate a division among the professional positions of the interviewees. While reporters were more critical about their effectiveness, high-level editors and managers demonstrated greater confidence in the mitigating measures. This is because journalists in higher echelons of Brazilian news organizations are more resistant to criticizing harmful statements in a context of high polarization. The following remark from a powerful media manager puts this dynamic into perspective:

You are not in the head of the politician. So, you cannot be certain that he is lying. Only in very few cases, when the information is well-known. But how do you prove intent? So, we need to be careful. We used the headline “President lies about” a few times, but very carefully. We decided not to engage in activism, like the *New York Times*. They lost their hand. It was “Trump lies” every day. (Journalist 36—Profile 3)

However, “mitigating measures” was the only theme with this kind of division. There was no substantial difference across the different types of participants, such as reporters, editors, and managers (i.e., profiles 1, 2, and 3), regarding their perceptions of problematic content and their rationales for publishing or not publishing such content. Interview data suggest that the final decision is heavily shaped by internal directives and senior staff in a more top-down news production process. These conditions seem to foster an environment that discourages challenges to such directives and reinforces agreement, corroborating previous studies that suggest journalists anticipate and adapt to both external and internal forms of pressure (Arndt 2018; Ferreira 2024b).

Overall, the perceptions and decision-making conveyed by interviewees indicate a pronounced pro-publication dynamic, thereby presenting a notable challenge to the democratic functions traditionally associated with legacy news media. A significant portion of the content was perceived as problematic precisely because of its potential capability to accentuate antidemocratic sentiments or erode support for democratic norms within public opinion. This dynamic holds far-reaching implications, particularly in a context of democratic instability or backsliding.

## Discussion and Implications

News media are crucial for democracy. However, journalists regularly find themselves in a dilemma concerning what to publish when dealing with content that may affect public opinion in a way that would hurt democracy. At the end of the day, the decision to publish a piece is guided in good part by journalists’ own norms and practices, and, more specifically, their expectations of the impact of problematic content on public opinion (e.g., hate speech, democratic attitudes, etc.). The scholarship so far has lacked a systematic analysis of journalists’ perspectives in this process. This is problematic given that they are central actors within this important democratic dilemma. In this article, we provided research findings about this missing perspective by using a qualitative approach that leveraged 36 semistructured in-depth interviews from journalists reporting on Brazil’s politics.

Our main findings are threefold: (1) Journalists do have opinions about what constitutes problematic content that would hurt democracy through public opinion. The two most important types of content that are viewed as problematic for public opinion were those that might undermine democratic attitudes as well as misinformation on important topics (e.g., COVID-19, climate change, etc.). (2) There was quite a strong consensus on the reasons why, in some instances, journalists should publish problematic content. Among other things, the notion of “competing voices,” the legitimacy that a position (e.g., president) gives to a person, the fear of being perceived as left leaning, and the pressure to keep one’s audience were key considerations. (3) There were also valid reasons to not publish problematic content. The reasons were focused on their negative impact on democratic attitudes, the failure of the media’s social and democratic role, and journalistic standards that might be interpreted as making it impossible to publish hate speech if one strictly adheres to ethical standards. However, some journalists aimed for a middleground between publishing problematic content and inserting mitigation measures to prevent hurting democratic attitudes and/or fueling hate.

These findings provide a more comprehensive and nuanced picture of the criteria used in journalists’ decision to publish or not to publish a story, particularly when their democratic role collides with notions of news values that structure the dynamics of gatekeeping theory. Indeed, our interview data suggest that the decision to publish problematic content prevails even if a majority of journalists see potential harm to public opinion and democratic norms. These discerned patterns within journalistic decision-making seem to be predominantly shaped by political and financial interests instead of the public interest. As such, we offer a more complex view of the mechanisms of influence in gatekeeping theory, advancing the understanding and theorization of these mechanisms.

The indication that journalists publish content they consider potentially harmful based on private interests is particularly problematic, given that prodemocratic functions of the news media are contingent upon editorial decisions guided in part by the public interest. These political and financial interests are reflected in those news organizations that have greater prominence and are usually enforced by media managers and high-level editors. However, it also accounts for the individual interests of journalists weighing perceptions, commitments, and constraints, often more severe among reporters and editors. Moreover, there is a lack of consistent criteria for publishing decisions and doubts regarding the effectiveness of mitigating measures. These somewhat muddled and permissive interactions in news productions seemingly create an environment where the publication of content that can negatively impact public opinion is normalized. This dynamic is particularly worrisome in countries undergoing democratic instability or backsliding since problematic content may risk damaging citizens’ democratic attitudes and eroding their trust in institutions against a backdrop of increased popularity for populist or authoritarian candidates.

## Supplementary Material

nfaf028_Supplementary_Data

## Data Availability

The interview transcripts underlying this article cannot be publicly shared in full to protect the identities of the participants. All participants were offered anonymity due to potential risks to their careers and personal safety (see Methodological Approach).

## Footnotes

1 “Democratic backsliding” (McDevitt 2022) or “de-democratization” (Bogaards 2018) refers to the process by which democratic institutions, norms, and practices are systematically weakened or dismantled by political actors, leading to a decline or reversal of a country’s previously achieved democratic conditions.

2 Bolsonaro served as a member of the Brazilian Parliament for 30 years and became a leader of a new far-right movement. Elected president in 2018, he was the first major candidate and sitting president to challenge democratic norms (Hunter and Power 2019).

3 V-Dem annually aggregates more than 450 indicators, including the integrity and fairness of elections, rule of law, separation of powers, accountability of executive power, political participation, and citizens’ rights and protections. The data are compiled into scores ranging from 0 to 1, where values closer to 1 indicate a higher quality of democracy. Brazil declined in key indexes of the project, such as Liberal Democracy (from 0.78 in 2015 to 0.53 in 2021) and Egalitarian Democracy (from 0.61 in 2015 to 0.38 in 2021).

4
Mason (2018, p. 58) defines “strategic sampling” in qualitative research as the process of producing, through sampling, a relevant range. In this process, she argues, the sample is designed to encapsulate a relevant range in relation to the wider universe, but not to represent it directly. As such, the researcher has a “strategic purpose in selecting a specified relevant range,” which means that “the relationship between the sample and the wider universe is not ad hoc, accidental, purely opportunistic or indeed representational” (Mason 2018, p. 58).

5 While snowball sampling is often critiqued for introducing potential bias, in this study, the supplementary use of this strategy did not introduce such bias. Rather, it facilitated the identification of key contributors in the news production chain who were not publicly credited. Suggestions from participants within the three selected media groups were specifically aimed at reconstructing the political teams of these organizations, thus seeking a balanced representation of different roles in the news production process. Furthermore, this approach did not introduce extraneous participants, as it was consistently employed to locate individuals already within our target group (journalists covering the 2018 election and Bolsonaro’s government).

6 Positionality is a central component of the process of qualitative data collection and analysis. This concept is broadly understood as where one stands in relation to “the other” in research (Merriam et al. 2001). It entails recognizing a researcher’s background and the position they choose to adopt within a study and in relation to participants (Savin-Baden and Major 2013).

7 In this section, we provided relevant and detailed examples of how we coded and interpreted our data, which are representative of the entire analysis process. However, we need to protect the confidentiality of the participants. Thus, including further examples and exposing large or specific portions of the interview transcripts would significantly increase the risk of identification.

8
*Petralhada* is a pejorative expression used in Brazilian politics to associate *petistas*, the supporters of the left-wing party PT, with the *Irmaos Metralha*, the Brazilian name of the Beagle Boys. The Beagle Boys are a group of cartoon characters created in 1951 by Carl Barks for Walt Disney’s Donald Duck universe. They are a family clan of organized criminals who always try to rob Scrooge McDuck.

9 For example, several newspapers not only published Bolsonaro’s statements perceived as racist or hate speech, but also published lists of his “best hits.” Among others, Folha de S. Paulo published the article “Bolsonaro Accumulates Prejudiced Phrases Against Different Targets; Remember” (Folha 2022).

## Appendix. AAPOR-Required Disclosure Elements

**First Data Source:** Transcripts of qualitative semistructured in-depth interviews conducted by one of the authors of the paper.

**Data Collection Strategy:** Semistructured with journalists reporting on Brazilian politics. Interviews were designed to explore journalists’ perceptions of public opinion, norms, and practices in the context of Brazil’s political landscape from 2018 to 2021. Rigorous follow-up questions were used to reduce post-hoc rationalization and overcome vague answers.

**Research Sponsor and Conductor:** There were no external sponsors. The research was self-initiated and conducted by the authors.

**Measurement Tools/Instruments:** Interview guide with open-ended questions and strategic follow-up questions. Questions were designed to elicit responses about journalists’ decision-making processes, the dilemmas they face, and their perceptions of problematic content. Follow-up questions were informed by previous studies (i.e., content analysis of news outputs published by the selected news organizations) and news outputs of the period of study. The interview guide is provided in the Supplementary Material.

**Population Under Study:** Journalists covering politics for the three major news organizations in Brazil (Globo, Record, and Folha/UOL) between 2016 and 2021. Participants included reporters, editors, and media managers with 10 to over 40 years’ professional experience. More characteristics cannot be disclosed to protect the identity of the participants.

**Methods Used to Generate and Recruit the Sample:** Open-ended desk research using the news coverage published between 2016 and 2021. Snowball techniques were later employed to identify journalists not normally credited in bylines (i.e., editors and managers). A target list of 81 journalists most involved in the political news coverage of this period was developed. Potential participants were contacted through email, cellphone, and social media like WhatsApp. They were assured anonymity and informed that interviews would contribute to knowledge production and improve our understanding of newsmaking, potentially leading to better journalistic practices. No other incentives were provided.

**Method(s) and Mode(s) of Data Collection:** 10 interviews were conducted in person in Brazil, and 26 were conducted via online video call systems, such as WhatsApp and Skype. Interviews lasted an average of 1.5 hours, and were conducted in Portuguese.

**Dates of Data Collection:** December 2021 to July 2022.

**Sample Sizes and Discussion of Precision:** A target list of 81 journalists was developed. A total of 36 journalists participated. There is no substantial difference between the average profiles of the target list and the participants (see the section Methodological Approach and Supplementary Material table S2).

**Whether and How the Data Were Weighted:** Not applicable.

**How the Data Were Processed and Procedures to Ensure Data Quality:** Interview data were transcribed and translated by the interviewer. Then, the authors conducted a thematic analysis, to systematically identify, organize, and interpret patterns (themes) within the interview data. Codes were developed using both deductive (theory-driven) and inductive (data-driven) strategies through multiple rounds of coding.

**Interviewer Details:** Interviews were conducted by one of the authors of the paper, who is a Brazilian former journalist with expertise in qualitative research and speaks Portuguese. No supervision or training was required since he was involved in all stages of the study.

**Study Stimuli:** No stimuli were used.

**Dispositions or Response or Participation Rates:** 44.4 percent (36/81).

**Sample Sizes:** The unweighted sample size for all analyses is 36 participants. The study targeted 81 journalists most involved in the political news coverage based on open-desk research of news archives, of which 36 participated, resulting in a 44.4 percent response rate. No weighting was applied as the sample is unweighted.

**Measurement and Model Specification:** Thematic analysis with a deductive-inductive coding process. See the section Analyzing the Data.

**General Statement Acknowledging Limitations of the Design and Data Collection**: Limitations include reliance on participants’ recollections of past events, potential post-hoc rationalizations, and potential bias from the interviewers. These were mitigated through rigorous follow-ups informed by news outputs and previous studies, and a process of active reflexivity conducted by the interviewer/authors during both data collection and analysis. See the section Conducting Interviews.
