# Supplementary material for: To Report or Not to Report? A Qualitative Analysis of Journalists’ Perspectives on Harm to Public Opinion
Source: Public Opin Q. 2025 Aug 7;89(SI):683–715. doi: 10.1093/poq/nfaf028 (PMC12411913; doi:10.1093/poq/nfaf028)
Supplement: nfaf028_Supplementary_Data [file nfaf028_supplementary_data.pdf]

Supplementary Material

"To report or not to report? A qualitative analysis of journalists' perspectives on harm to public opinion"

Ricardo R. Ferreira, Teaching Assistant, School of Social and Political Science, University of Edinburgh, Edinburgh, Scotland, 0000-0003-2926-4678*

Jean-François Daoust, Assistant Professor, School of Applied Politics, Université de Sherbrooke, Sherbrooke, QC, Canada, 0000-0002-2736-7430

** Corresponding author*:
School of Social and Political Science, University of Edinburgh
Chrystal Macmillan Building - 15a George Square
Edinburgh, Scotland, EH8 9LD
ricardo.rf@ed.ac.uk

Table of contents

Supplementary Text: Interview Questions 2

Supplementary Tables 6

Table S1. Sample status 6

Table S2. Profile variation 6

Supplementary Text: Interview Questions

The interview questions and the data set collected with them are part of a larger project analyzing the role of journalists and news organizations during a democratic decline in Brazil. The interview guide was designed to understand how journalists covered key political events in the country between 2016 and 2021 (i.e., Operation Car Wash, 2018 Election Campaign and Jair Bolsonaro's Presidency). This broad aim was then broken down into more specific interview questions. Then, the questions and possible follow-ups were adapted to the unique aspects of each case study to ensure the collection of valuable data within the project's focus and parameters of commonality for cross-case analysis.

Therefore, the interview guide aimed at exploring journalists' rationale during news production and the chain of decisions in their newsrooms for all news outputs pertaining to the news coverage of the selected political events. This included decisions regarding what to publish or not and their perceptions of the value and risks for public opinion posed by such publications. However, we avoid leading questions to increase the validity of our data. Instead of including direct questions about definitions and publication criteria of problematic content, we designed broad questions on production practices, decisions and editorial meetings to minimize journalists' rationalizations. Finally, we designed and included more specific follow-up questions regarding the publication dilemma in anticipation of this paper, which were also informed by the news content published by the selected news organizations in the period of our study.

For this research, we focused on the data related to the 2018 presidential election campaign (January to October 2018) and the first three years of Jair Bolsonaro's government (2019 to 2021). As such, we provide the interview guide for case studies 2 (2018 Election Campaign) and 3 (Jair Bolsonaro's Presidency) below.

Case 2 – 2018 Election Campaign

1. Now, think about the news coverage of the 2018 presidential campaign. What do you remember?

> *Possible follow-ups:*
> *How did journalists approach it?*
> *What do you think about the coverage?*

2. How fair do you think the coverage was?

> *Possible follow-ups:*
> *There are indications that journalists were soft on Bolsonaro. What do you think of that?*
> *(cross-check with news content)*

3. How did you (or your colleagues) decide to tell the stories related to the election?

> *Possible follow-ups:*
> *Were there any specific concerns?*
> *(cross-check with news content)*

4. What conversations did people have during the editorial meetings (briefs meetings and edition meetings)?

Possible follow-ups:
I wonder if any specific instructions stand out for you.
How were these received/carried out?
(cross-check with news content)

5. Two leading candidates: one was associated with disinformation and authoritarian discourse, and the other was connected to a recent corruption condemnation. How do you address these challenges during an election campaign coverage?

Possible follow-ups:
Were there any concerns about scrutinizing the right amount and balancing the coverage?
(cross-check with news content)

6. During the coverage of the presidential campaign, what did you think about your role (the role of the press)?

Possible follow-ups:
Did these thoughts affect the work?

7. Take another trip with me. Think about one particular day during the campaign or a story from the election period that marked you and you truly remember. Travel back to that day. How were the editorial decisions made that day/for that story? Walk me through this day/this work.

Possible follow-ups:
How did you decide what to pursue or not?
What to highlight or not? Who to talk to or not?

8. What did each person contribute?

Possible follow-ups:
Was anyone left out?

9. What sort of things did influence what you told me about (the work during this coverage)?

Possible follow-ups:
What kind of things enabled/helped, and what constrained/got in the way of the work during those times?
Can you tell me more about what was tricky?

Case 3 – Jair Bolsonaro's Presidency

1. Now, think about the news coverage of the first years of Bolsonaro's government. What do you remember?

> *Possible follow-ups:*
> *How did journalists approach it?*
> *What do you think about the coverage?*

2. How fair do you think the news coverage about the government is?

> *Possible follow-ups:*
> *Some news outlets are facing criticism for being too negative (or too positive) towards the government. What do you think of that?*
> *(cross-check with news content)*

3. In the US, some newspapers saw the Trump administration as a different challenge, which led to different 'rules of engagement' and even extra investments to prepare for the coverage. I wonder how it was with Bolsonaro.

> *Possible follow-ups:*
> *Tell me more about that. How did you describe such complex situations as attacks on the press, Covid-19, and corruption cases?*

4. What conversations did people have during the editorial meetings (briefs meetings and edition meetings)?

> *Possible follow-ups:*
> *I wonder if any specific instructions stand out for you.*
> *How were these received/carried out?*
> *(cross-check with news content*)

5. I'm curious. Do you think the news coverage of Bolsonaro changed after the election and became more negative (or positive)? You would say that?

> *Possible follow-ups:*
> *Has it changed, has the press changed, or is it possible that we missed something in 2018?*
> *(cross-check with news content)*

6. Now, choose one particular story or day you strongly remember during the Bolsonaro government. Last trip, I promise. Choose a day or story that marked you. How were the decisions that day?

Possible follow-ups:
How did you decide what to pursue or not?
What to highlight or not? Who to talk to or not?

7. What did each person contribute?

Possible follow-ups:
Was anyone left out?

8. What sort of things did influence what you told me about (the work covering the government)?

Possible follow-ups:
What kind of things enabled/helped, and what constrained/got in the way of the work during those times?
Can you tell me more about what was easy/tricky?

Supplementary Tables

Table S1. Sample status

Interviewed online (i.e., video calls)	26
Interviewed in-person	10
Replied and denied	13
Initially agreed but stopped replying	10
Never replied	22
Total target	81

Table S2. Profile variation

**Professional position**	**Interviewed**	**Non-Interviewed**
Profile 1: Reporters and TV producers	21	27
Profile 2: Editors, Assistant Editors, or similar job details	12	14
Profile 3: Media managers (executive positions such as Editor-executive, Editor-in-chief, or Newsroom Director	3	4
Total	36	45
